# *Euglena gracilis* Promotes *Lactobacillus* Growth and Antioxidants Accumulation as a Potential Next-Generation Prebiotic

**DOI:** 10.3389/fnut.2022.864565

**Published:** 2022-06-22

**Authors:** Junjie Dai, Jiayi He, Zixi Chen, Huan Qin, Ming Du, Anping Lei, Liqing Zhao, Jiangxin Wang

**Affiliations:** ^1^College of Chemistry and Environmental Engineering, Shenzhen University, Shenzhen, China; ^2^Shenzhen Key Laboratory of Marine Bioresource and Eco-Environmental Science, Shenzhen Engineering Laboratory for Marine Algal Biotechnology, College of Life Sciences and Oceanography, Shenzhen University, Shenzhen, China

**Keywords:** *Euglena gracilis*, prebiotics, antioxidants, *Lactobacillus*, metabolomics

## Abstract

*Euglena gracilis*, a single-celled microalga with various trophic growth styles under different cultivation conditions, contains nutrients, such as ß-1,3-glucans, essential amino acids, fatty acids, vitamins, and minerals. It has recently attracted attention as a new health food. Among them, ß-1,3-glucans, paramylon of *Euglena*, is an insoluble dietary fiber and is well known as an immune booster, attenuator of obesity and diabetes, reducer of acute liver injury, and suppressor of atopic dermatitis, and other chronic inflammatory disorders. Recently, evidence has appeared for the positive health effects of foods, food ingredients, or biochemical compounds derived from several other microalgae, such as *Chlorella*, *Spirulina*, *Dunaliella*, *Phaeodactylum*, and *Pavlova*. Until most recently, the prebiotic activity of *Euglena* and paramylon was reported. Emerging prospects of microalgae as prebiotics were well summarized, but the mechanisms behind the bacterial growth promotion by microalgae are not elucidated yet. Thus, we evaluated the prebiotic prospects of both autotrophic and heterotrophic *Euglena* on six different *Lactobacillus*. What’s more, the stimulated mechanism was revealed by bacterial culture medium metabolomic analysis. This study could widen the knowledge about the prebiotic activity of *Euglena* as a next-generation prebiotic and other microalgae-derived compounds as potential health foods.

## Introduction

The prebiotic concept was first defined in 1995 (1) as “a nondigestible food ingredient that beneficially affects the host by selectively stimulating the growth and/or activity of one or a limited number of bacteria in the colon, and thus improves host health.” In 2016, the International Scientific Association for Probiotics and Prebiotics updated the definition of a prebiotic (2): a substrate that is selectively utilized by host microorganisms conferring a health benefit. The nondigestible oligosaccharides fructans and galactans are the prebiotics most extensively documented (3, 4). Meanwhile, a fraction of dietary fiber and dietary polyphenols have real potential as emerging prebiotics (5–7).

*Euglena gracilis* is a unicellular microalga lacking a cell wall with both autotrophic and heterotrophic growth styles under different cultivation conditions. *Euglena* cells contain many nutrients, such as ß-1,3-glucans, tocopherol, carotenoids, essential amino acids, vitamins, and minerals, and have recently attracted attention as a new health food (8). These products have antioxidant, antitumor, and cholesterol-lowering effects (9). Among them, ß-1,3-glucans, paramylon of *Euglena*, is an insoluble dietary fiber and is well known as an immune booster (4), the attenuator of obesity and diabetes (2, 3), reducer of acute liver injury (10), suppressor of atopic dermatitis, and other chronic inflammatory disorders (5). Consequently, the use of *Euglena* as healthy food is promising.

Recently, evidence has appeared for the positive health effects of foods, food ingredients, or biochemical compounds derived from several other microalgae, such as *Chlorella*, *Spirulina*, *Dunaliella*, *Phaeodactylum*, and *Pavlova* (11). Until recently, the prebiotic activity of *Euglena* and paramylon was reported (12, 13). For example, *Euglena* stimulates *Faecalibacterium* in the human gut and contributes to increased defecation (12). Heterotrophic *Euglena* cell extract and isolated paramylon led to the growth promotion of Lacfid (*Lactobacillus*) (13). However, whether any other substances in *Euglena’s* potential as prebiotics remain unknown. Emerging prospects of microalgae as prebiotics were well summarized, but the mechanisms behind the bacterial growth promotion are not elucidated yet.

To verify the prebiotic potential of *Euglena*, we selected *Euglena* powders derived from two different culture styles autotrophic and heterotrophic cultivation. Six commercial probiotic *Lactobacillus* (14) were selected to evaluate the possible prebiotic activity of *Euglena*. Growth and antioxidant activity were measured. The results showed that both *Euglena* powders (AE and HE) could promote growth, and the antioxidant properties of *Lactobacillus* were also improved. Interestingly, green *Euglena* powder has a better promotion effect even though yellow *Euglena* powder contains more paramylon. We successfully investigated 1,398 metabolites of culture medium to find nitrogen metabolism, biosynthesis of amino acids, and biotin metabolism pathways instead of paramylon improved bacterial growth under *Euglena* addition. This study could widen the knowledge about the prebiotic activity of *Euglena* and other microalgae-derived compounds as potential health foods.

## Materials and Methods

### Materials and Chemicals

The *Euglena gracilis* CCAP 1224/5Z powders (autotrophic *Euglena* as “AE,” heterotrophic *Euglena* as “HE” afterward) were supplied by Yougetiancheng Biotechnology Co., (Yiwu, China) (15). The nutrients in the *Euglena* powders, such as paramylon, total proteins, lipids, amino acids, vitamins, and minerals, were determined using standard methods. 2,2-Dipheny1-1-picrylhydrazyl (DPPH) and Pyrogallic acid were purchased from Shanghai Aladdin Biochemical Technology Co., Ltd. (Shanghai, China). All other chemicals were purchased from commercial suppliers and of analytical grade.

### Bacterial Species

Six probiotics, *Lactobacillus hordei* (MRS 102), *L. kefiri* (MRS 103), *L. brevis* (MRS 104), *L. parabuchneri* (MRS 107), *L. buchneri* (MRS 108), and *L. fructivorans* (MRS 109), were achieved from Liqing Zhao laboratory of Shenzhen University (14). The probiotics were stored in MRS broth containing 25% glycerol at −80°C.

### Effect of *Euglena* Powders on the Growth of Probiotic Bacteria

Six probiotics were employed to investigate the effect of *Euglena* powders on prebiotic activity. The green *Euglena* powder AE and yellow powder HE were separately added to MRS broth (30 ml) at the final concentrations of 0.1, 0.2, 0.5, 1.0, and 3.0% (w/w). The mixtures were then 5.0% inoculated and cultured overnight culture of a lactic acid bacteria strain and incubated with shaking at 200 rpm at 30°C and sampled in triplicates at different time points (0, 4, 6, 8, 12, and 24 h).

Meanwhile, the sample was diluted and plated onto the full MRS agar plates to determine viable bacterial count (cfu/ml). Based on results from our preliminary experiments, 1.0% Euglena powder additions, 12 and 24 h as logarithmic and platform growth phases were chosen for bacterial growth evaluation. The MRS medium without *Euglena* powder was used as the control group for metabolomic analysis.

### *In vitro* Antioxidant Analysis

The overnight culture of *Lactobacillus* was centrifuged at 1,000 × *g* for 5 min at 4°C to separate the insoluble *Euglena* powder. Then the resulting supernatant was harvested and centrifuged at 7,000 × *g* for 15 min at 4°C. Finally, the cell pellet was washed thrice with PBS (pH 7.2). The antioxidant analysis of *Lactobacillus* was evaluated using DPPH free radical scavenging ability and superoxide anion free radical scavenging ability.

A total of 2 ml cell suspension and 2 ml of 0.2 mmol/L ethanolic solutions of DPPH radical were mixed and incubated at room temperature for 30 min in the dark. After incubation, the reaction mixture was centrifuged at 7,000 × *g* for 15 min at 4°C, and the absorbance at 517 nm was measured. The mixture of DPPH and sterile distilled water served as the blank sample. The mixture of DPPH and *Euglena* powder was used as the control group. The scavenging ability was defined as follows:


$$\mathrm{Scavenging}\mathrm{ability}\left(\%\right)=\left[1-A_{517\,\left(s\,a\,m\,p\,l\,e\right)}/A_{517\,\left(b\,l\,a\,n\,k\right)}\right]\times 100\%$$

The first ml of cell suspension was mixed with 3 ml of Tris–HCl buffer (pH 8.2). And the sterile distilled water was used as the blank sample. Then, 1 ml pyrogallol solutions (1.2 mmol/L) were added, and the autoxidation activity was evaluated by measuring the absorbance at 325 nm after incubation for 25 min. The following formula calculated the scavenging ability:


$$\mathrm{Scavenging}\mathrm{ability}\left(\%\right)=\left[1-A_{325\,\left(s\,a\,m\,p\,l\,e\right)}/A_{325\,\left(b\,l\,a\,n\,k\right)}\right]\times 100\%$$

### Metabolic Analysis

Species MRS 104 showed the most apparent promotion growth with the additions of EA and HE was selected for further metabolomic analysis. The bacterial media during logarithmic growth was collected for metabolic analysis. The metabolites in the sample were extracted and analyzed according to the method mentioned by He et al. (16). Each sample group included four biological replicates. Metabolites were extracted from bacterial solution using the method of Doppler. LC–MS/MS analyses were performed using a UHPLC system (Vanquish, Thermo Fisher Scientific) with a UPLC BEH Amide column (2.1 mm × 100 mm, 1.7 μm) coupled to Q Exactive HFX mass spectrometer (Orbitrap MS, Thermo). The mobile phase consisted of 25 mmol/L ammonium acetate and 25 ammonia hydroxide in water (pH = 9.75) (A) and acetonitrile (B). The autosampler temperature was 4°C, and the injection volume was 3 μl. The QE HFX mass spectrometer was used for its ability to acquire MS/MS spectra on information-dependent acquisition (IDA) mode in the control of the acquisition software (Xcalibur, Thermo). To include as many metabolites as possible, both Electron Spray Ionization (ESI) positive and negative ion modes data were collected. The metabolomic data were log2-transformed and then processed using principal component analysis (PCA) from MetaboAnalyst. It summarizes the contribution of each variable to the model. The metabolites with VIP > 1 and *p* < 0.05 (Student’s *t*-test) were significantly changed. In addition, commercial databases, including the KEGG database^1^ and MetaboAnalyst^2^, were used for metabolic pathway enrichment analysis. From these analyses, bubble diagrams and metabolic pathways were made.

### Statistical Analysis

All the experiments were conducted in triplicate. The experimental results were presented as the mean ± SD. Significant analysis was analyzed by one-way ANOVA. All the tests were performed using SPSS statistical software (version 16.0, SPSS Inc., United States).

## Results

### Nutrients Inside AE and HE and in *Euglena* Added Culture Medium

As summarized in Table 1, noticeable content differences in AE and HE were observed, especially for paramylon, proteins, and lipids. High content of paramylon (70.1% w/w), low proteins (22.2%), and lipids (3.4% w/w) were detected in HE, while contents of proteins and lipids in AE were much higher, 55.1 and 24.5%, respectively. Similarly, higher contents of Fe, Zinc, linolenic acid, and linoleic acid in AE were also detected in this study. According to amino acids, all have higher ranges in AE than those of HE, almost with two-folds.

**TABLE 1:** Nutrient contents in different *Euglena* powders, AE and HE as auto- and heterotrophic *Euglena*, respectively.

Bioactive molecules	Heterotrophic (mean ± SD)	Autotrophic (mean ± SD)	Unit	Assessment method
Paramylon	70.1 ± 5.6	11.3 ± 2.83	g/100g	SDS-HCl
Proteins	22.2 ± 8.4	55.1 ± 5.82	g/100g	GB 5009.5-2016
Total lipids	3.4 ± 0.82	24.5 ± 2.31	g/100g	GB 5009.6-2016
ASP	1.4 ± 0.05	2.54 ± 0.7		
THR	0.76 ± 0.12	1.35 ± 0.2		
SER	0.64 ± 0.44	1.1 ± 0.1		
GLU	1.96 ± 0.23	3.64 ± 0.39		
GLY	0.78 ± 0.02	1.6 ± 0.8		
ALA	1.27 ± 0.2	3.08 ± 1.03		
VAL	1.06 ± 0.47	1.93 ± 0.66		
MET	0.31 ± 0.02	0.51 ± 0.01		
ILE	0.64 ± 0.04	1.18 ± 0.05	g/100g	GB 5009.124-2016
LEU	1.33 ± 0.1	2.52 ± 0.87		
TYR	0.47 ± 0.02	0.9 ± 0.27		
PHE	0.77 ± 0.09	1.38 ± 0.18		
LYS	0.85 ± 0.04	1.83 ± 0.02		
HIS	0.28 ± 0.11	0.52 ± 0.018		
ARG	1 ± 0.06	1.61 ± 0.12		
PRO	0.91 ± 0.08	1.75 ± 0.07		
Total amino acids	14.4 ± 2.27	27.4 ± 5.49	g/100g	
Vitamin E	16.9 ± 1.32	10.6 ± 2.34	mg/100g	GB 5009.82-2016
Fe	70.9 ± 6.8	294 ± 12.9	mg/kg	GB 5009.268-2016
Ca	69.9 ± 11.2	71.8 ± 8.38	mg/kg	
Zinc	19.2 ± 0.8	71.5 ± 12.1	mg/kg	
Mg	2.9 ± 0.39	1.13 ± 0.8	g/kg	
DHA	0.0449 ± 0.0012	0.0289 ± 0.001	g/100g	GB 5009.168-2016
Linolenic acid	0.0473 ± 0.0184	0.125 ± 0.03	g/100g	GB 5009.168-2016
Linoleic acid	0.0797 ± 0.001	0.116 ± 0.07	g/100g	GB 5009.168-2016
Carotenoids	0.144 ± 0.082	0.64 ± 0.08	g/Kg	
Unsaturated fatty acids	2.37 ± 1.29	2.26 ± 1.32	g/100g	GB 5009.168-2016
Pb	<0.04	<0.04	mg/Kg	GB 5009.12-2017
Cd	<0.02	0.061	mg/Kg	GB 5009.15-2014
Hg	<0.05	<0.05	mg/Kg	GB 5009.17-2014
Arsenic	<0.04	0.19	mg/Kg	GB 5009.11-2014
Bacteria	270	<10	CFU/g	GB 4789.2-2016
*E. coli*	<0.3	<0.3	MPN/g	GB 4789.3-2016
*Salmonella*	<0.3	<0.3	MPN/g	GB 4789.4-2016
*Shigella*	n/a	n/a	/25g	GB 4789.5-2012
*Staphylococcus*	n/a	n/a	/25g	GB 4789.10-2016
Mold	<10	<10	CFU/g	GB 4789.15-2016

As shown in the differentially expressed metabolomic data (Supplementary Table 1, POS groups) at the “positive ion” mode, among top 45 metabolites with > 100 fold increase in AE+ culture medium compared to that of the control 33 (73.3%) are “lipids and lipid-like molecules,” and relatively low number (total 29 metabolites > 100-fold increase) detected in HE+ samples 19 are lipids. They are PC(14:0/14:0), LysoPC(20:4(5Z,8Z,11Z,14Z)), PC(14:0/15:0), LysoPC(20:2(11Z,14Z)), LysoPC(22:4(7Z,10Z,13Z,16Z)), PC(18:3(9Z,12Z,15Z)/18:1(11Z)), PC(20:1(11Z)/14:0), PC(18:1(11Z)/14:0), etc. (Supplementary Table 1), most of them are unsaturated fatty acids.

Under the “negative ion” mode, 18 of 19 TOP metabolites with > 100-fold increase in the AE+ culture medium are “lipids and lipid-like molecules,” such as adrenic acid, hypogeic acid, ginsenoside B2, sciadonic acid, leukotriene B4, 15S-hydroxy-5Z,8Z,11Z,13E, 17Z-eicosapentaenoic acid, and hepoxilin B3 in AE+ sample; 13/13 metabolites with > 100-fold increase are “lipids and lipid-like molecules” in HE-treated culture medium, namely, adrenic acid, eicosadienoic acid, sciadonic acid, ginsenoside B2, arachidonic acid (ARA), leukotriene B4, docosapentaenoic acid (22n-3) (DPA), hypogeic acid, myristoleic acid, alpha-tocopherol, hepoxilin B3, and eicosapentaenoic acid (EPA) (Supplementary Table 1, NEG groups).

The contents of lactic acid, isobutyric acid, and dodecanoic acid in different samples were studied (Supplementary Table 2). Similar levels of lactic acid, the relative abundance of 349.2 and 337.85 based on metabolomics data, were also detected in both AE and HE powder controls (AE+MRS, HE+MRS), lower than those in bacterial only (MRS103, MRS104). When AE and HE were added to MRS103, lactic acid in the co-cultured medium was reduced if combined lactic acid contents (as [AE+MRS103] vs. [AE+MRS] + [MRS103]) were considered. To MRS104, the addition of AE showed no significant change, while HE increased lactic acid content significantly.

Autotrophic *Euglena* (AE) and HE have similar contents of isobutyric acid with a little higher level than that in MRS103 or MRS104 only. The addition of AE or HE into MRS103 and MRS104 caused an obvious reduction of isobutyric acid (as [AE+MRS103] vs. [AE+MRS] + [MRS103]) (Supplementary Table 2). The final level was higher in AE added bacteria (AE+MRS103 and AE+MRS104) than in HE addition (HE+MRS103 and HE+MRS104). The comparative contents of dodecanoic acid showed low contents (i.e., 0.06) in bacteria (MRS103 and MRS104) and high levels in AE and HE (1.89 and 2.53). Thus, AE and HE provided a high level of dodecanoic acid in the coculture medium. Only HE addition resulted in a significant combination decrease in the coculture medium (Supplementary Table 2). Other fatty acids showed similar differential changes in the treatments.

### Both *Euglena* Powders Stimulate All *Lactobacillus*

**FIGURE 1:**
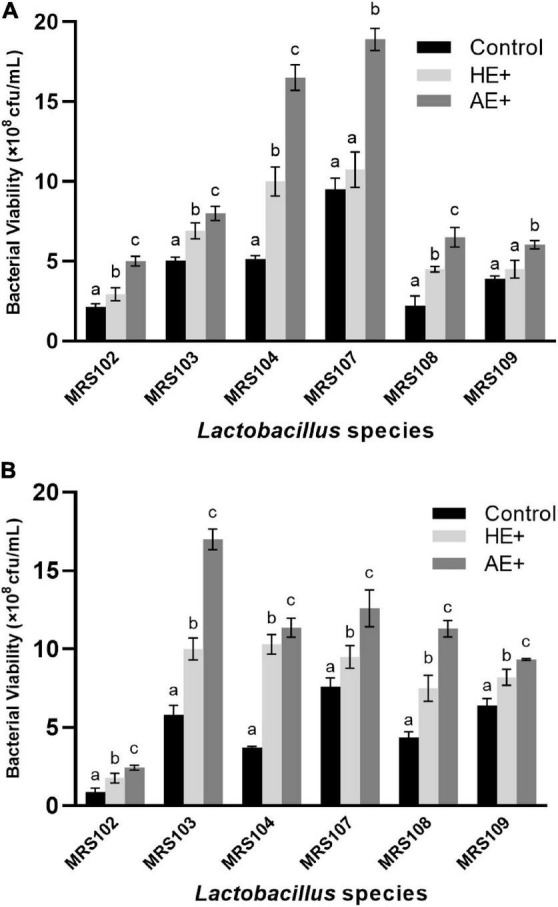
The counting of viable bacteria during logarithmic growth **(A)** and platform stages **(B)** of six selected *Lactobacillus* strains on the addition of *Euglena* powders (AE+ and HE+).

The bacterial growth was first investigated with normal OD600 absorption, however, both 0.2 and 1% addition of *Euglena* powders showed obvious absorption in the culture medium (Supplementary Figure 1). We used a dilution plate on the full MRS agar plates to determine the viable bacterial count (cfu/ml) in each sample. The counting of viable bacteria during logarithmic growth and platform stages of six selected *Lactobacillus* on *Euglena* powders (AE and HE) was shown in Figures 1A,B, respectively. Different *Lactobacillus* showed different growth patterns, MRS102 with low cell numbers at both growth stages, while MRS104 and 105 reached high cell numbers at the log phase and MRS103 increased cell numbers until the platform stage. All selected *Lactobacillus* grew well in the presence of both *Euglena* powders. Compared with controls, in the presence of *Euglena* powders, especially AE, the numbers (by up to three-fold) of *Lactobacillus* were significantly increased. Also, the growth-promoting effect of AE is superior to that of HE. At the platform stage, while some *Lactobacillus* cell viability decreased, the addition of AE or HE promoted bacterial growth at an early stage.

### *Euglena* Enhanced Bacterial Antioxidant Activity

**FIGURE 2:**
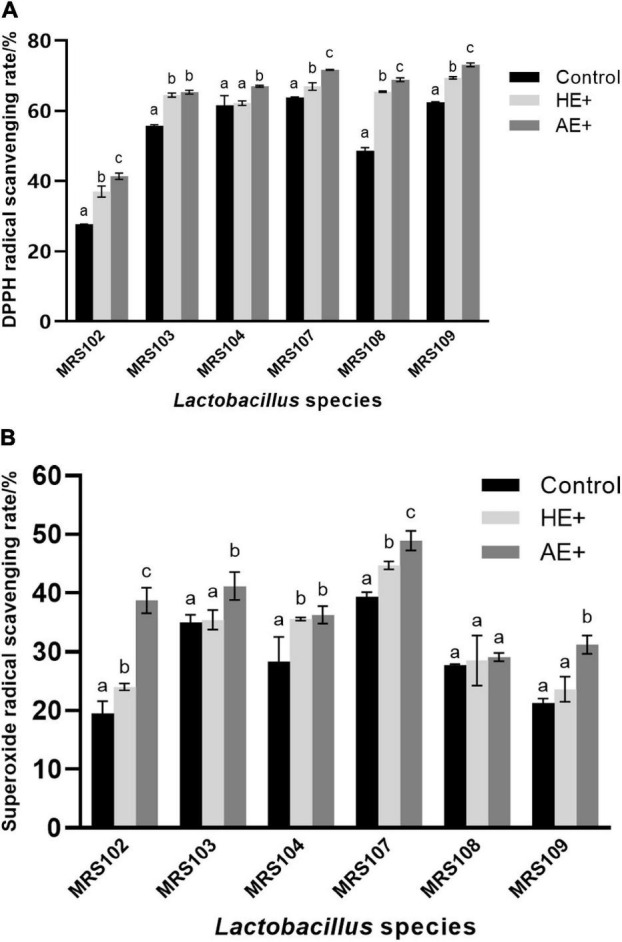
The antioxidant analysis of *Lactobacillus* strains was evaluated using DPPH free radical scavenging ability **(A)** and superoxide anion free radical scavenging ability **(B)** on the addition of *Euglena* powders (AE+ and HE+).

The antioxidant potential of *Lactobacillus* plays a vital role in protecting the host microflora from the attack by free radicals. The six selected *Lactobacillus* revealed solid antioxidant activity against free radicals, as shown in Figure 2. In the presence of *Euglena* powder, the antioxidant activity of *Lactobacillus* was significantly improved. And the effect of green *Euglena* powder is superior to the yellow one. The results demonstrated that the *Euglena* powder could improve significant antioxidant activity, and the AE is better than HE.

### *Lactobacillus* Culture Medium Metabolic Profiles

**FIGURE 3:**
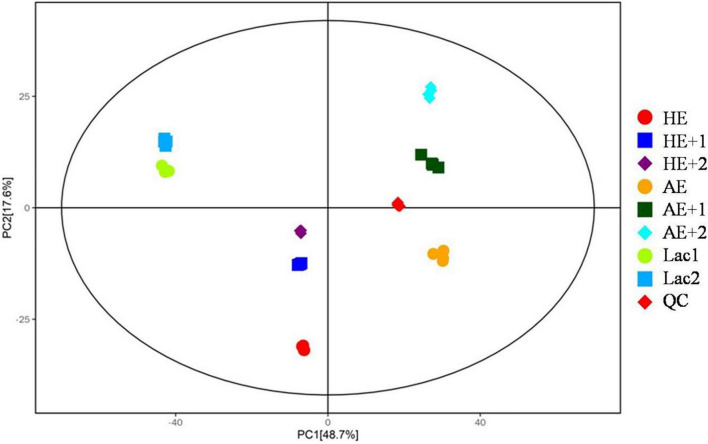
Principal component analysis (PCA) analysis of metabolomic profiles. HE and AE the powders only in MRS medium without bacteria; HE+1, HE+2, AE+1, AE+2, and replicates of addition of HE and AE into the bacterial medium with bacteria; Lac1 and Lac2 replicates of bacterial in MRS medium only as control; and QC, technical control.

After data processing, the total metabolic features were obtained in positive and negative modes. PCA was conducted to see significant metabolomic differences before and after adding *Euglena* powder. The result is shown in Figure 3. Each group in both models showed good separation, and all samples were nearly within a 95% CI.

This study detected a total of 1,011 and 387 metabolites in our bacterial culture media under positive and negative ion modes. According to the selection criteria (i.e., VIP score > 1 and *p* values < 0.05 in Student’s *t*-test), 218 and 196 matched differential metabolites were identified in positive and negative modes between HE and the control group. A total of 399 and 191 matched differential metabolites were identified between AE and the control (Table 2). The addition of *Euglena*, either AE or HE, provided more metabolites (shown as upregulated metabolites, Table 2), especially in HE (negative ion mode) with 187 upregulated metabolites, including pyruvate, succinate, L-aspartate, L-glutamate, nicotinate, and biotin, with only nine metabolites downregulated when compared with controls.

**TABLE 2:** Metabolomic data based on a culture medium with the addition of auto- and heterotrophic *Euglena*, AE+ and HE+, respectively.

	Ion type	Up	Down	Total differentially	Total detected
AE+ vs. control	POS	189	210	399	1011
	NEG	114	77	191	387
HE+ vs. control	POS	147	71	218	1011
	NEG	187	9	196	387

*Numbers in the table are up, down, total differentially regulated, and total detected numbers of metabolites.*

**FIGURE 4:**
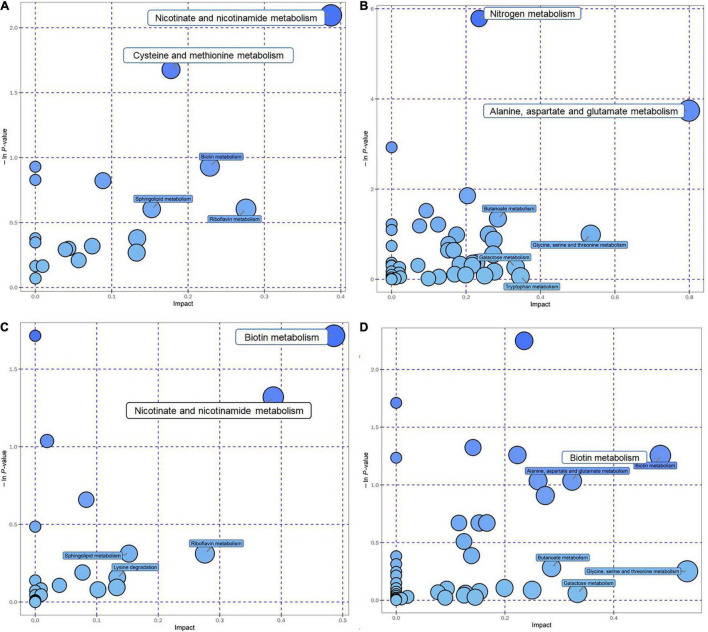
The top KEGG pathways of groups with the addition of AE and HE vs. control are presented in the bubble chart. Each bubble in the bubble chart represents a metabolic pathway. The *X*-axis of the bubble and the bubble scale indicates the influence factor of the pathway in the topology analysis. The larger the size, the greater the influence factor; the *Y*-axis indicates the enrichment analysis. *P*-value [take the negative natural logarithm, namely, log10(*p*)]. **(A)** HE+(POS); **(B)** HE+(NEG); **(C)** AE+(POS); and **(D)** AE+(NEG).

After the KEGG database annotated these metabolites, critical metabolic pathways were screened according to their position and role in the relevant metabolic pathways (Supplementary Table 2). A total of six pathways were selected and regarded as the key ones with the addition of AE and HE, namely, metabolic pathways, biosynthesis of secondary metabolites, ABC transporters, purine metabolism, cysteine and methionine metabolism, and biosynthesis of amino acids (Supplementary Table 2). According to the bubble chart, critical metabolic pathways related to HE addition are nicotinate and nicotinamide metabolism, cysteine, and methionine metabolism (positive mode, Figure 4A) and nitrogen metabolism, and alanine, aspartate, and glutamate metabolism (negative mode) (Figure 4B). In contrast, several key metabolic pathways are relevant to AE: biotin metabolism and nicotinate and nicotinamide metabolism in the positive mode (Figure 4C); and biotin metabolism in the negative mode (Figure 4D).

## Discussion

In recent years, evidence has appeared for the positive health effects of foods, food ingredients, or biochemical compounds derived from microalgae (11). With microalgae, some probiotic bacteria showed improved growth, health product yield, and other desirable properties. For instance, adding a green microalga *Chlorella vulgaris* at 0.1–1.5% dose shortens the log phase, improves lactic acid yield and enzyme activity, and acidifies probiotic *Lactobacillus brevis* (17). Supplementing *C. vulgaris* and a cyanobacterium *Spirulina platensis* increased the viability of probiotic lactic acid bacteria in milk or yogurt products and also the sensory attributes (18–20). As feed for animals, *S. platensis* helped absorb metal ions in the animal gut to restore gut disorders due to imbalance of insulin and adipose distributions (21), and improved lactic acid bacteria viability and activity. They suppressed the growth of pathogenic bacteria, thus improving the host’s adsorption (22). A marine diatom *Phaeodactylum tricornutum* improved the animal immune system and increased intestinal adsorption (23) and *Dunaliella tertiolecta* improved the immune system. They enhanced immune system disease in shrimps (24). Moreover, *Navicula sp.* addition improved the immune system and enhanced antioxidant properties (25). Similar to *S. platensis*, other microalgae, such as *Chlorococcum*, *D. salina*, *S. magnus*, and *Chlorella*, also stimulate the growth of lactic acid bacteria possible by xylose and galactose from the microalgal extract was also reported (26).

As an animal feed, *Euglena* could promote the growth of probiotic *Streptococcus iniae*, offering immunostimulant activity to red drum fish (27). It also improved animal hosts’ health and immune system by enhancing *Bacillus licheniformis* or *B. subtilis* (28). In a recent report, *Euglena* stimulates *Faecalibacterium* in the human gut and contributes to increased defection (12). In addition, the prebiotic activity of paramylon isolated from heterotrophic *Euglena* was reported to lead to cell number enhancement of Lacfid (*Lactobacillus*) (13). After 36 h cultivation, a significant difference in *Lactobacillus* growth was obtained by adding *Euglena* powder and paramylon individually, showing a ∼25% increase in cfu per ml of *Lactobacillus* compared to that of control (13). This study investigated nutrients from AE and HE based on different culture methods. Both *Euglena* powders significantly improve bacterial growth and antioxidants production after 12 and 24 h addition, with some differential promotion patterns to individual *Lactobacillus* strains. Thus, *Euglena* whole cells could be a potential next-generation prebiotic. Compared with other reported prebiotic microalgae, *Euglena* shows a significant advantage: it is devoid of the cell wall to make its relatively high biological accessibility rate (97.3% in the human gut), which means the contents inside *Euglena* cells are highly accessible.

Microalgal biomass comprises a wide range of bioactive compounds, such as protein, polysaccharides, pigments, vitamins, fatty acids, and minerals (29–31). The promising prebiotic compounds are polysaccharides and their derivatives, such as exopolysaccharides, fucoidans, alginates, and carrageenans. They are not entirely fermented by colonic microbiota and act as prebiotics. However, growth promotion and performance of probiotics by prebiotic microalgae are not limited by these compounds directly, and such enhancements are also reported indirectly, such as suppression of pathogens, removing toxic substances, improving gut adsorption, improving disease resistance and immunity, enhancing their viability, and storage (11).

Though dozens of reports showed microalgal prebiotic activity, no metabolomic analysis was employed to study the metabolite changes in the bacterial culture medium so far. In this study, reliable metabolomic data indicated that the addition of *Euglena* powders involves increased metabolism, biomaterial transportation, purine metabolism, and amino acid biosynthesis pathways, with vital metabolic pathways like biotin metabolism, nicotinate and nicotinamide metabolism, and nitrogen metabolism. With significantly higher contents of amino acids, AE probably provided more biomaterial, such as amino acids for bacterial growth and metabolism. Prebiotic soluble fibers are fermented by beneficial bacteria in the colon to produce short-chain fatty acids, which are proposed to have systemic anti-inflammatory effects (32). We also explored the relations of selected short-chain fatty acids with prebiotic growth. AE and HE provided a relatively high level of dodecanoic acid in the coculture medium. Considering a much higher level of total lipids in AE, and more significant prebiotic activity in bacteria, we proposed that fatty acids in *Euglena* may contribute to bacterial growth promotion. What’s more, like biotin, many lactic acid bacteria require increased biotin for growth in media lacking aspartate (33), and *Euglena* powders provided amino acids and biotin to promote bacterial growth.

Approximately 70–80% of the carbohydrate content was paramylon as detected in our lab and previous reports (34), and paramylon is a water-insoluble linear (unbranched) β-(1,3)-glucan polysaccharide polymer with a molecular mass of about 500 kDa (35). Interestingly, there are no carbon or polysaccharides metabolism pathways enriched or differentially expressed with the addition of either AE or HE (with 70.1% paramylon), which suggests that at least paramylon may not be the key or significant bacterial promotion factor in this case. Perhaps other carbohydrates and other chemicals promote, which could be further investigated later.

Except for ß-glucan, several other critical bioactive compounds derived from microalgae showed relatively fewer prebiotic properties (11), such as steroids, carotenoids, fatty acids, lectins, minerals, vitamins, amino acids, and polyketides (36). Thus microalgae are promising sources of the majority of the above compounds. Few of them are already verified to possess prebiotic attributes (37). *Euglena* produces essential amino acids, minerals, unsaturated fatty acids, and vitamins E in AE and HE, with higher amino acids, fatty acids, and vitamins in AE than HE. More effective bacterial growth promotion and antioxidants enhancement in AE added bacterial culture was observed in this study. Considering there is much less paramylon (11% in AE vs. 70.10% in HE) and much higher contents of lipids, primarily unsaturated fatty acids, in AE inside *Euglena* cells and culture medium in this study. It is reasonable to propose that unsaturated fatty acids in *Euglena* cells and culture medium may also contribute to prebiotic activity in *Lactobacillus* strains. Furthermore, elaborately designed experiments would be conducted to verify this proposal.

With the apparent higher prebiotic activity of AE than HE, we proposed that paramylon may not be the only or even primary prebiotic bioactive molecules in *Euglena* to promote *Lactobacillus* growth. Our result is consistent with the findings that other compounds in *Euglena* instead of paramylon stimulate the growth of *Faecalibacterium* since paramylon did not stimulate the bacterial growth *in vitro* (12). The prebiotic potential of soluble β-1,3-glucan from cereals and mushrooms is well documented (38, 39). However, long-chain nondigestible paramylon prebiotic activities are largely unknown. This study verified the prebiotic activities of bioactive compounds other than paramylon in *Euglena* powders. We first reported that autotrophic *Euglena* has a higher prebiotic activity than heterotrophic *Euglena* cells in *Lactobacillus* strains. This study provides insight into improving its prebiotic activity as animal feed and functional food for humans. Further investigation into the effects of *Euglena* cells and paramylon *in vitro* and *in vivo* on probiotics and humans will be required to better evaluate its probiotic growth promotion activity as a novel prebiotic.

## Data Availability Statement

The original contributions presented in this study are included in the article/Supplementary Material, further inquiries can be directed to the corresponding authors.

## Author Contributions

JW and JH conceived and designed the experiments. JD, HQ, and MD performed the experiments. JD and ZC analyzed the data. JD wrote the manuscript. AL, ZC, and JW revised the manuscript. All authors read and approved the final manuscript.

## Conflict of Interest

The authors declare that the research was conducted in the absence of any commercial or financial relationships that could be construed as a potential conflict of interest.

## Publisher’s Note

All claims expressed in this article are solely those of the authors and do not necessarily represent those of their affiliated organizations, or those of the publisher, the editors and the reviewers. Any product that may be evaluated in this article, or claim that may be made by its manufacturer, is not guaranteed or endorsed by the publisher.

## Footnotes

1
http://www.genome.jp/kegg/

2
http://www.metaboanalyst.ca/

## Supplementary Material

Supplementary Table 1 Differentially expressed metabolites summary and metabolites details under AE+ vs. control, HE+ vs. control, at both negative (NEG) and positive (POS) ion modes.

Supplementary Table 2 KEGG pathways enriched under AE+ vs. control, HE+ vs. control, at both negative (NEG) and positive (POS) ion modes.
