# Supplementary material for: Euglena gracilis Promotes Lactobacillus Growth and Antioxidants Accumulation as a Potential Next-Generation Prebiotic
Source: Front Nutr. 2022 Jun 22;9:864565. doi: 10.3389/fnut.2022.864565 (PMC9257220; doi:10.3389/fnut.2022.864565)
Supplement: Supplementary Table 2 — KEGG pathways enriched under AE+ vs. control, HE+ vs. control, at both negative (NEG) and positive (POS) ion modes. [file Table_2.DOCX]

**Supplementary Table S2. Contents of lactic acid, isobutyric acid, and dodecanoic acid in different samples.** The relative abundance of each chemical was expressed as mean ± standard error based on metabolomic data. In the same column, we used the sum of AE+MRS and MRS103 as the control, and compared AE+MRS103 and HE+MRS103 with individual controls, respectively. The same is true for MRS104. The green and red color highlight the reduced or increased trends compared with the control.

| Groups | Lactic acid | Isobutyric acid | Dodecanoic acid |
| --- | --- | --- | --- |
| AE+MRS | 349.20±3.55 | 6.02±0.10 | 1.89±0.09 |
| AE+MRS103 | **987.53±28.57^**^** | **4.98±0.15^***^** | 1.68±0.10 |
| AE+MRS104 | 1581.23±62.64 | **4.79±0.18^***^** | 1.86±0.07 |
| HE+MRS | 337.85±6.17 | 5.92±0.09 | 2.53±0.12 |
| HE+MRS103 | **963.97±50.51^*^** | **4.41±0.28^***^** | **1.90±0.24^*^** |
| HE+MRS104 | **1809.51±78.94^**^** | **4.42±0.22^***^** | **1.80±0.11^**^** |
| MRS103 | 830.23±21.83 | 4.43±0.14 | 0.06±0.01 |
| MRS104 | 1090.78±22.90 | 4.34±0.10 | 0.06±0.01 |

* indicates *p*< 0.05; ** indicates *p*< 0.01; *** indicates *p*< 0.001.
