# Supplementary figures and images for: Euglena gracilis Promotes Lactobacillus Growth and Antioxidants Accumulation as a Potential Next-Generation Prebiotic
Source: Front Nutr. 2022 Jun 22;9:864565. doi: 10.3389/fnut.2022.864565 (PMC9257220; doi:10.3389/fnut.2022.864565)

## Slide 1
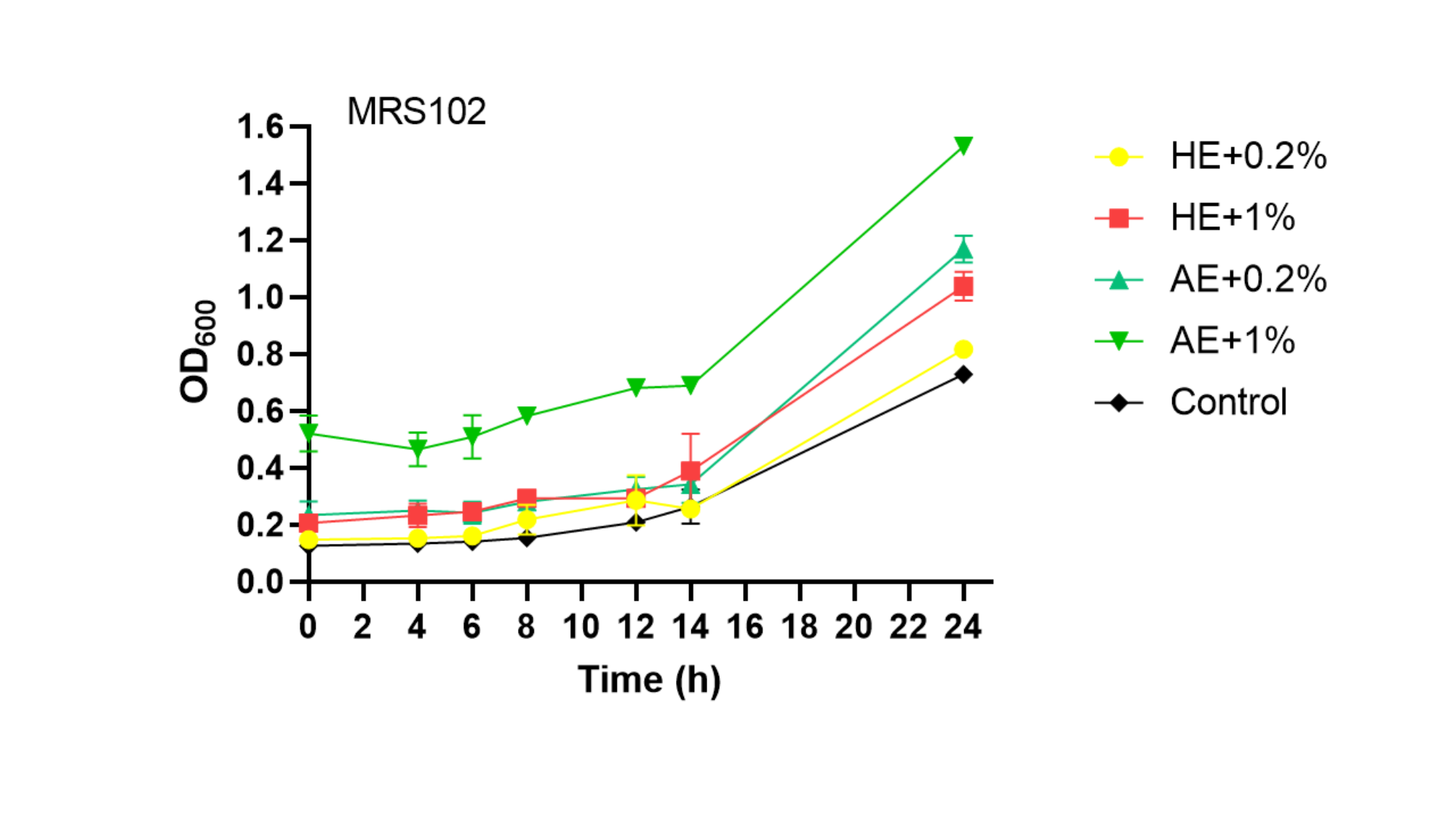

## Slide 2
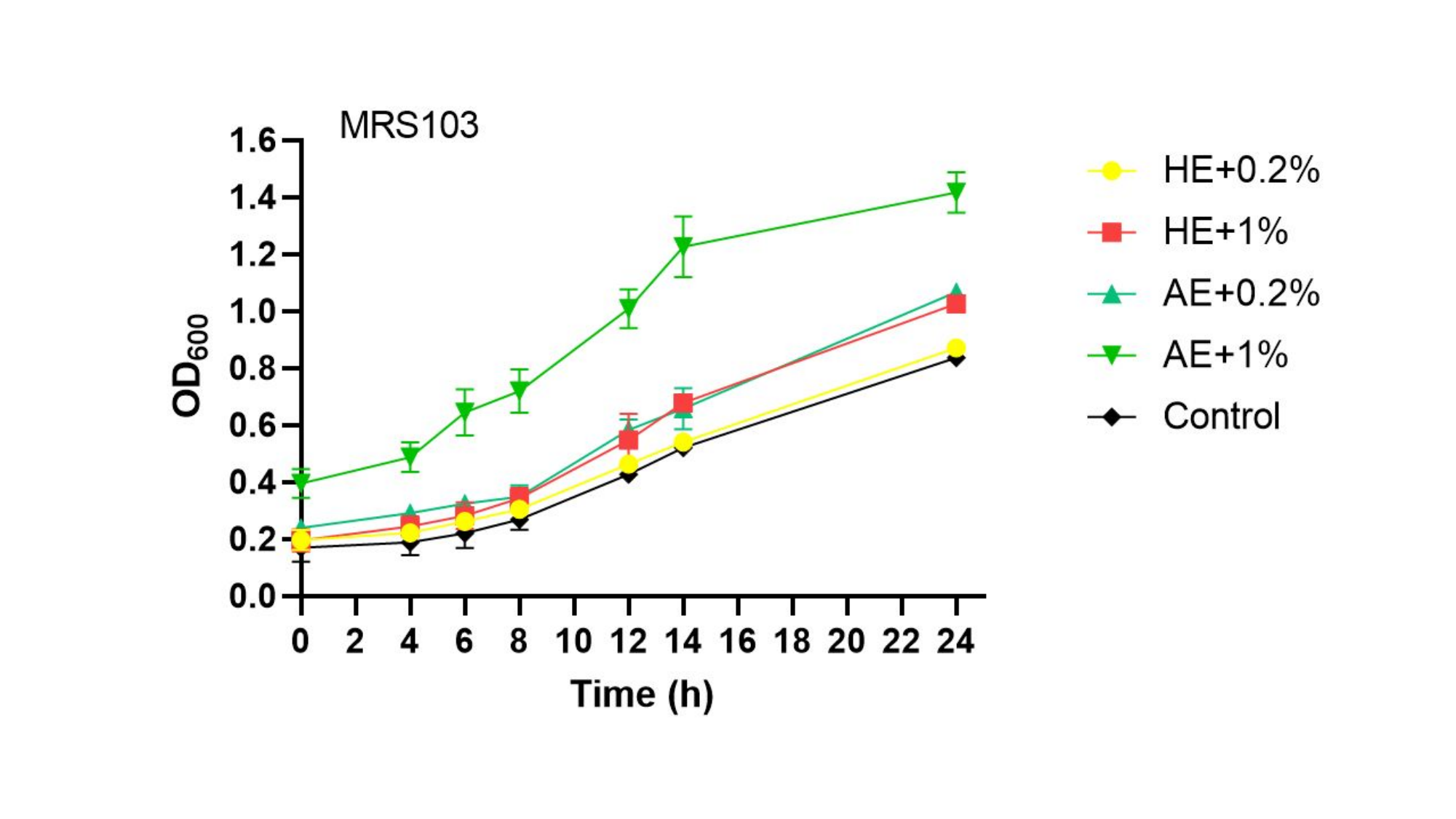

## Slide 3
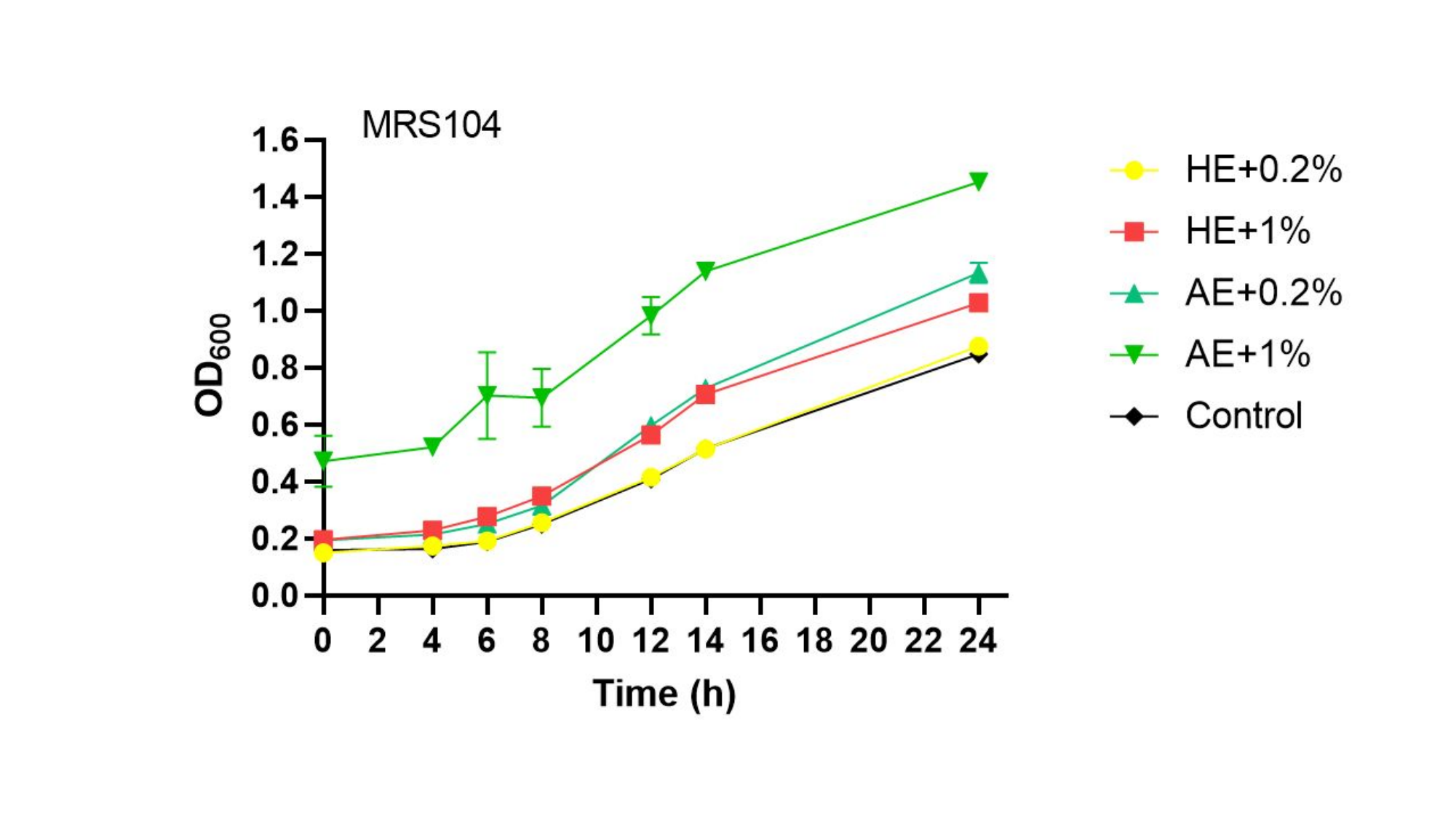

## Slide 4
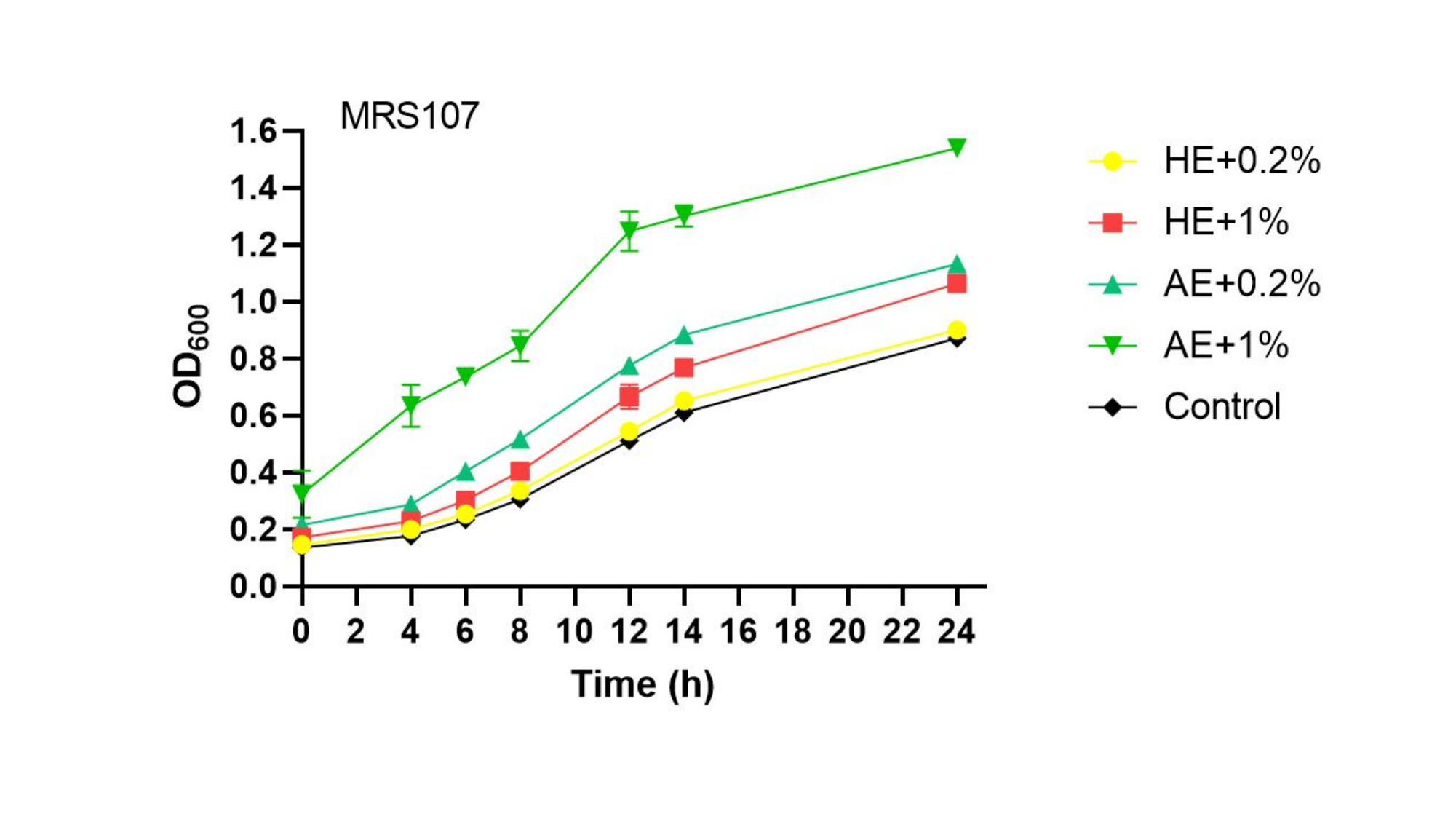

## Slide 5
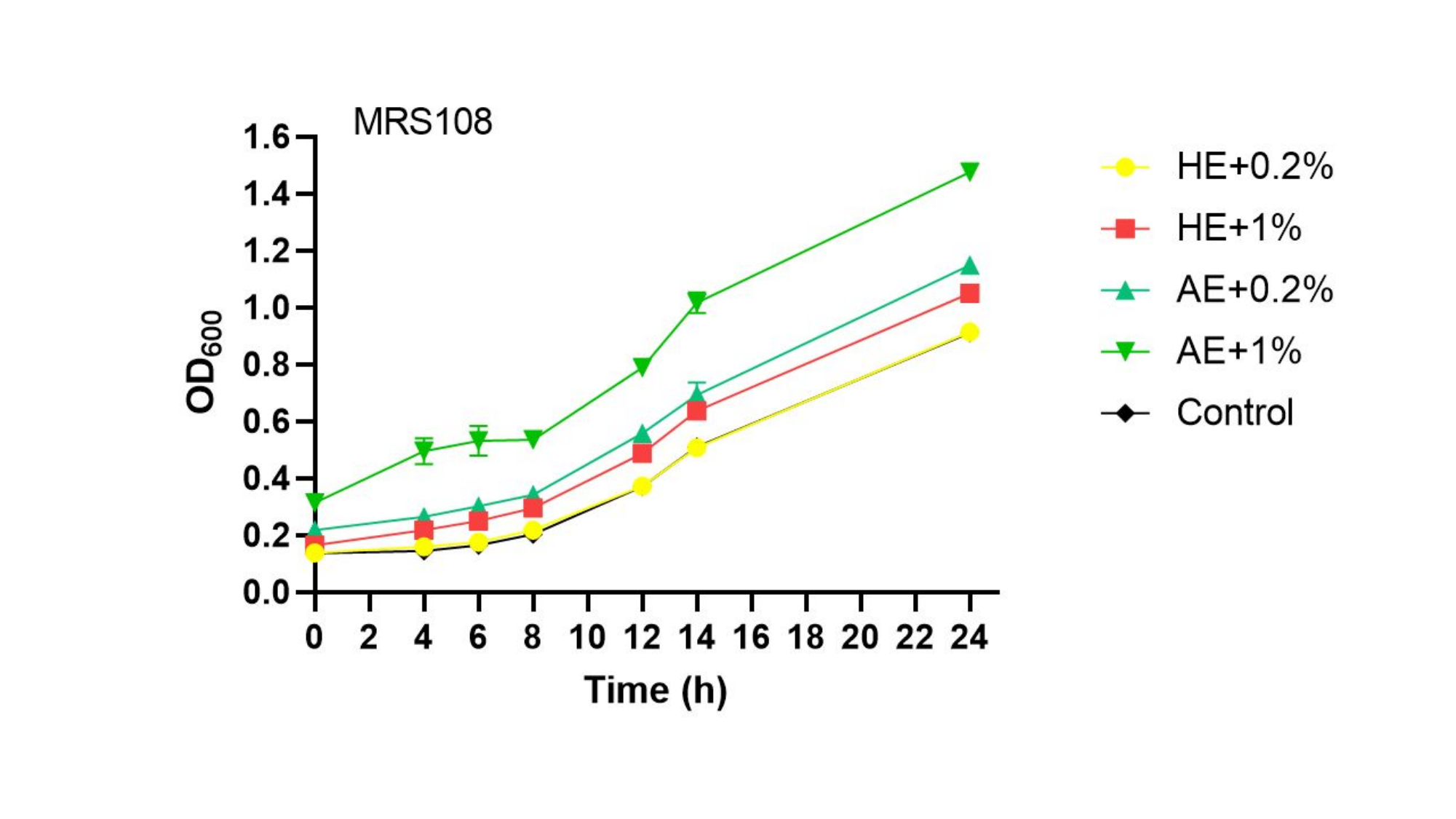

## Slide 6
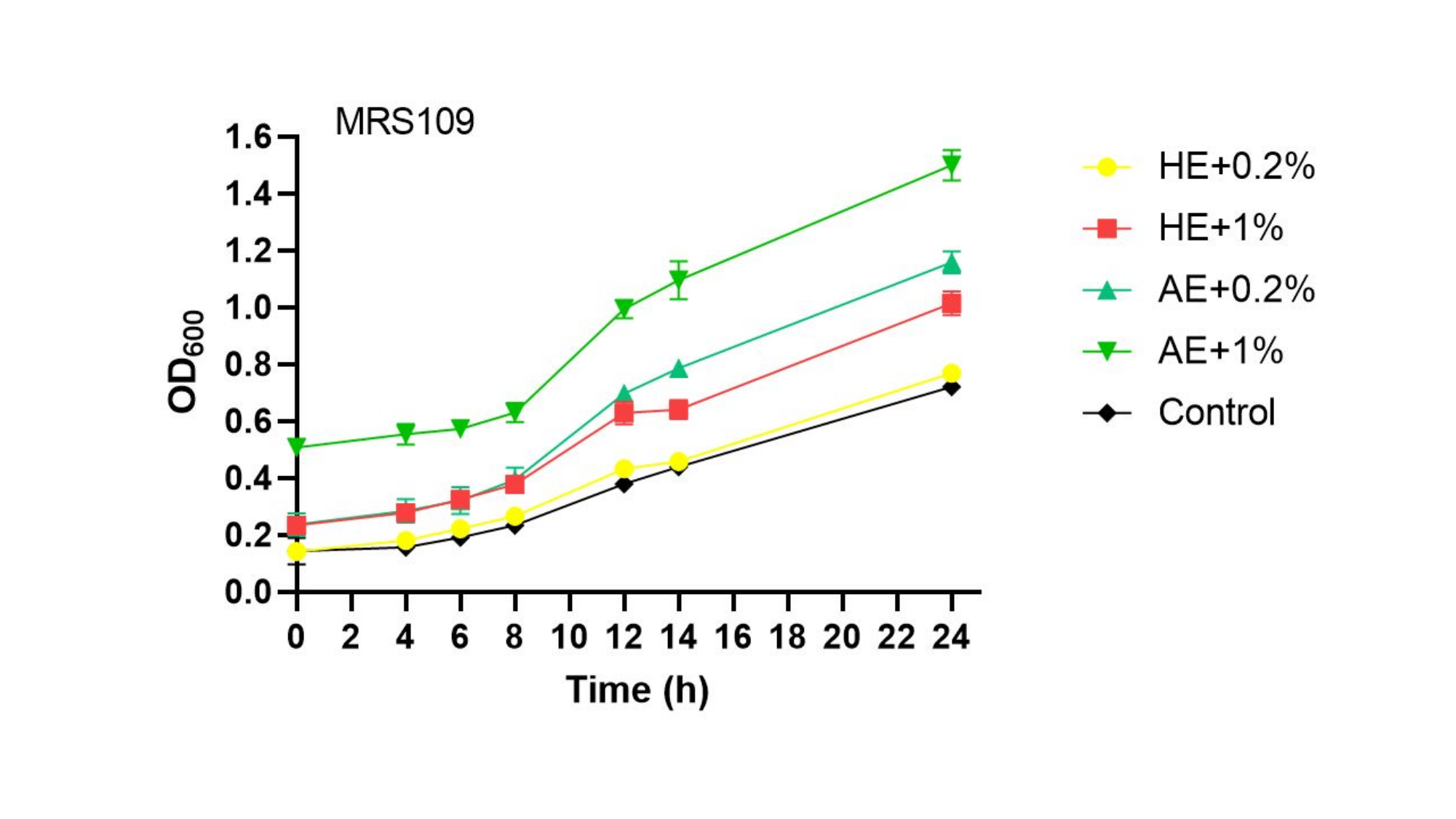

Supplement: Supplementary file 4 [file Presentation_1.PPTX]
